# Supplementary material for: A self-adaptive hardware with resistive switching synapses for experience-based neurocomputing
Source: Nat Commun. 2023 Mar 21;14:1565. doi: 10.1038/s41467-023-37097-5 (PMC10030830; doi:10.1038/s41467-023-37097-5)
Supplement: Supplementary file 1 — Supplementary Information [file 41467_2023_37097_MOESM1_ESM.pdf]

A SELF-ADAPTIVE HARDWARE WITH RESISTIVE SWITCHING SYNAPSES FOR EXPERIENCE-BASED NEUROCOMPUTING

Supplementary material

This supplementary material includes technical information about the memory devices used in this work and over the implemented experimental setup. It also proposes further analysis referred to the bio-inspired recurrent neural network.

The whole information of this document deals with the theory and hardware realization of experience-based learning systems in the framework of reinforcement learning, Supplementary Fig. 1(a). In particular, it is here proposed a resilient system able to act on memristive weights (RRAM devices, used for connecting the neurons and for mapping the internal states) in order to achieve autonomous navigation in a continually changing environment, Supplementary Fig. 1(b).

The first section of this document deals with the fabrication and the electrical characterization of the resistive switching memory devices (RRAM). The second section presents the whole experimental setup in detail, constituted by (i) a System-On-Chip (which acts as master), (ii) the SiO_x RRAM arrays, and (iii) the technical equipment for the programming of the devices. Then, further sections are dedicated to the simulation studies of the behaviour of the agent as a function of the environmental evolution and to the benefits introduced by non-volatile memories in comparison with CMOS-based approaches in terms of scalability and reconfigurability. A final part of this supplementary information is also dedicated to the modelling of the bio-inspired recurrent neural network and to the mathematical relation between the reinforcement parameters and circuital variables.

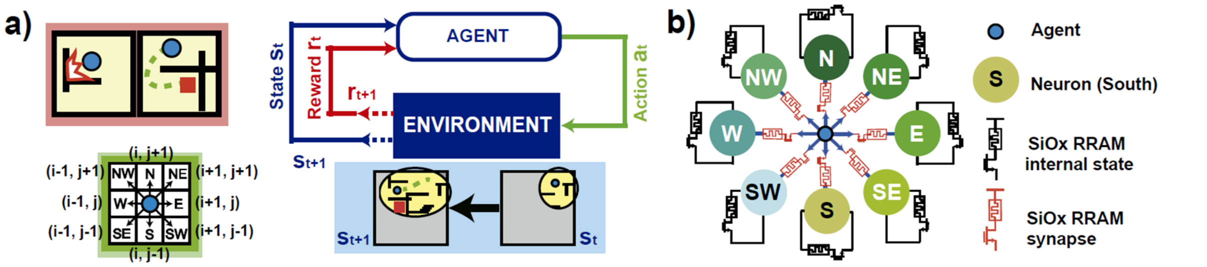

Supplementary Figure 1. High level representation of memory-based autonomous navigation by reinforcement learning. (a) An agent (e.g., a robot) moves in an environment (e.g., a maze) and receives penalties and rewards as a result of decision-making operations. For instance, hitting a wall gives rise to a penalty, while finding the red square gives rise to a reward. (b) Scheme of the main RRAM synaptic connections between the current positions and the nearest neighbours. The synaptic connections are potentiated or depressed according to the Hebbian Learning theory. Note that each direction of movement of the agent has a specific internal state which is also configured by using the RRAM devices.

DISCUSSION OVER THE ELECTRICAL CHARACTERIZATION OF THE DEVICES

The reinforcement learning algorithm has been verified by software simulations in MATLAB environment. The algorithm uses experimental resistance data obtained from the electrical characterization of the devices. In particular, the characterization process has been focused on the study related to the resistive window, at low and high programming frequencies, and to the multilevel capability of the devices. Concerning this last point, we modulated the gate voltage V_G of the transistor in series to the cell (1T1R configuration) to obtain different levels of compliance current I_C, hence different values of low resistive state (LRS). We also modulated the stop voltage, V_{STOP} in order to obtain a resistive modulation of the high resistive state (HRS) of the devices, Fig. 1 of the manuscript.

The forming process of the devices and the consequent DC characterization were carried out using an HP4156C parameter analyser controlled by MATLAB. Supplementary Fig. 2(a) shows some superimposed examples about the forming of the conductive filament of the resistive switching devices. The forming has been performed by applying a triangular waveform at the top electrode (TE) of the cells. During forming, the peak voltage generally varies from 3V to 4V. The subsequent DC characterization data have been obtained applying a positive peak voltage of 2.5V at the TE for the set operation. The higher the gate voltage of the transistor (from 1.5V to 2.5V), the higher the I_C, the lower the LRS. The reset process from LRS to HRS of the SiO_x RRAM devices presented in Supplementary Fig. 2 is obtained by exchanging top and bottom electrodes (BE) and applying a peak voltage (V_{STOP}) between 1.3 V (lower HRS) and 1.6 V (higher HRS). The script is modulable, and the user determines the number of cycles per gate voltage, for different values of V_{STOP}, Supplementary Fig. 2 (b-c).

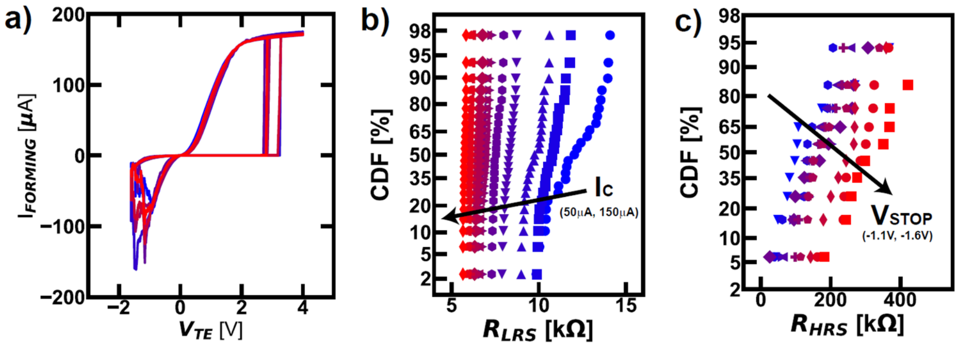

Supplementary Figure 2. Electrical characterization of the RRAM devices. (a) Forming characteristics of SiO_x RRAM devices. (b) LRS distribution after forming the devices using different compliance current I_C. (c) HRS distributions after forming as a function of the stop voltage V_{STOP}.

DISCUSSION OF THE EXPERIMENTAL SETUP

The experimental setup used to test the system and to study the role of the synaptic devices consists of several memristor arrays, a Xilinx SoC (System on chip, a low-cost commercial Zynq 7000), an 8-channel oscilloscope with low-noise probes (Tektronix MSO58), a pulsed signal unit (TTI TGA12104) and a desktop computer, which was used to keep track of the evolution of the system and for debugging purposes. In the following, a detailed information regarding the connectivity of the setup, the use of the experimental equipment and the operating mode are provided. A low-noise custom printed circuit board was realized using AutoCad Eagle. The PCB board is able to host the wire-bonded memory arrays, Supplementary Fig. 3(a), and it is directly connectable to the Zynq 7000, Supplementary Fig. 3(b). Several chips with RRAM arrays have been used in the experimental setup, Supplementary Fig. 3(c), in order to implement the whole system for reinforcement learning tasks.

The following subsections include technical information about the procedures for the development of this research study, including the used devices and the experimental setup.

Architecture of the integrated hardware

Every state array is separated from the synaptic array and each of them has a dedicated direct-memory-access (DMA) circuit addressable by means of the pads reported in Supplementary Fig. 4. For simplicity, given the high number of available devices, the state and synaptic arrays have been addressed using different arrays with same dimensions, but this choice is arbitrary: for instance, changing the conceptual architecture, it could have been also possible to use the same array (i.e., sharing the same DMA). Furthermore, note that the array is built following a general-purpose setup and offers both fully connected architectures (for the implementation of matrix-vector-multiplication) as well as stand-alone addressable devices for single-ended functionality and electrical characterization. Note also that the maximum dimension of the array that can be accessed using only one DMA is around 16kb (128x128), while the smallest fully connected array addressable by the hosting board and the experimental setup shown in Supplementary Fig. 3(a) has a dimension of 8x8. Finally, note that the high reconfigurability of these arrays gives the possibility of choosing different top-level architectures for taking advantage of different features of the devices, depending on the application and target.

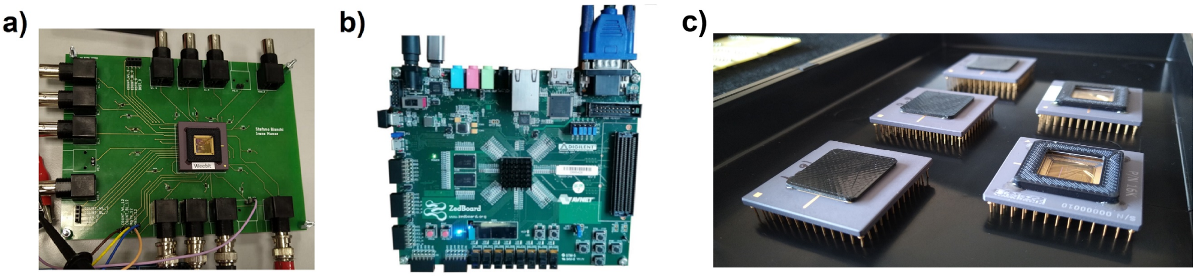

Supplementary Figure 3. Sketch of the main blocks of the experimental setup. (a) Printed circuit board hosting the arrays of RRAM synaptic elements. (b) Xilinx Zynq 7000, the low-cost SoC (System on chip) used in the experimental setup as master of the system. Several peripherical connections were also provided towards the desktop computer and the measuring equipment for debugging purposes. (c) Test chips with RRAM arrays used for the experimental measurements.

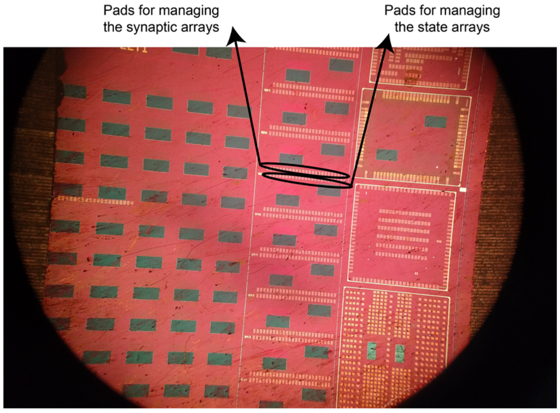

Supplementary Figure 4. Photo at the optical microscope highlighting the "Synaptic" and "State" arrays available in the fabricated hardware with the corresponding programming pads.

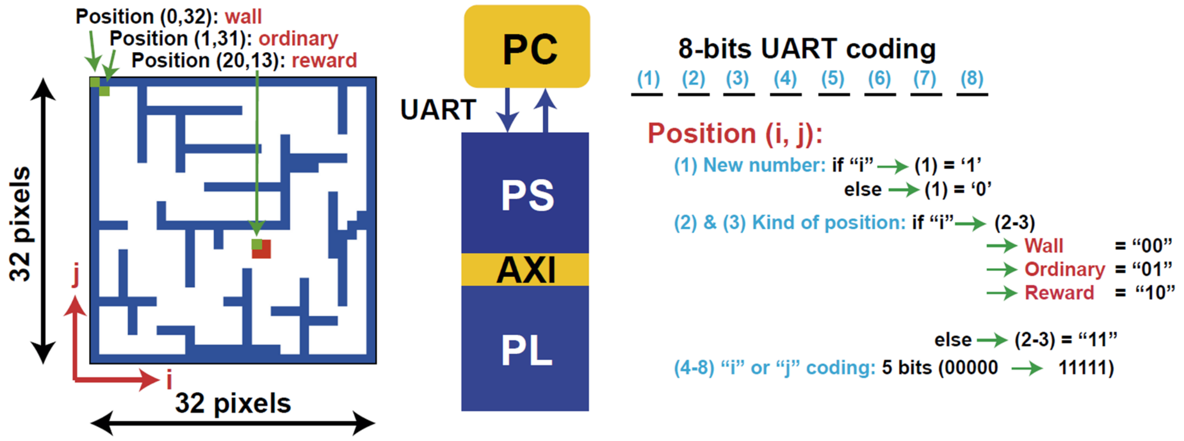

Supplementary Figure 5. Scheme of the UART connection for receiving/transmitting the positions of the maze. The 8 bits used for the UART protocol are coded for communicating not only the location in the maze ("i" row and "j" column) but also for including additional information, such as the type of the position, and for debugging purposes. Such architecture enables reusability and reconfigurability of the environment.

Data transmission to the SoC

The experimental setup is constituted by (i) a Xilinx Zynq-7000 SoC (System on Chip); (ii) several SiO_x-RRAM arrays; (iii) a computer executing MATLAB; (iv) a TTI TGA12104 arbitrary waveform generator to set, partially set, or reset the memory cells; (v) an HP HP4156C semiconductor device parameter analyser; (vi) a Tektronix MSO58 oscilloscope with 8-channels.

The SoC acts as master of the system and manages the SiO_x RRAM arrays. It has two main blocks: (i) the Processor System (PS), consisting of a dual core ARM Cortex-A9, and (ii) the Programmable Logic (PL), which is an FPGA Series 7. This SoC is mounted in the Zedboard, a low-cost development PCB. The SoC also acts as interface with the external world, since the user can directly act on it to design the maze to be tested. Thus, the SoC both maps the environment and drives the solution by implementing the reinforcement learning algorithm with respect to the RRAM devices. Note that the choice of implementing an electronic environment is done to mimic the real-world events where an autonomous agent lives environmental experiences. In particular, the autonomous maze navigation to study the learning by reinforcement has been drawn from biological studies[1].

As master, the SoC reads the spikes coming from the eight neurons that map the eight possible directions along which the agent can move, i.e., the eight fundamental cardinal points. The spiking activity in time determines the successive positions covered by the agent, thus electronically mapping the movement inside the maze. This movement is not completely free, e.g., the agent cannot go through the walls (it receives a penalty). Furthermore, the agent must record the last positions if the final goal is achieved (reward). The SoC knows the maze configuration decided by the user in order to determine the programming state of the devices: when a wall is hit, the corresponding internal state is re-programmed as a LRS synapse, thus with a high neuronal threshold; conversely, if the agent receives the reward, the RRAM synapses are set to LRS for Hebbian learning, and the ones that determine the threshold states are programmed in HRS, thus lowering the threshold of the rewarded neurons. This algorithm mimics what actually is observed in biological experiments with mice[1]. It can also be seen as a hardware simulation of the sensorial exploration of a maze by a robot that autonomously builds its own policy thanks to bio-inspired reinforcement learning.

The external user defines not only the dynamic maze (as 32x32 matrices), but even the rate of moving walls, the total number of trials, and the number of movements per each trial. This information must be known by the SoC to properly control the peripherals of the system. Therefore, a communication line between the SoC and the computer has been defined using the UART (Universal Asynchronous Receiver/Transmitter) connection, Supplementary Fig. 5.

The UART sends and receives serial data through two wires: a receiving wire, RX, and a transmitting wire, TX. UART transmitted data are organized into packets formed by the start bit (equal to '1'), the data frame (the actual data transferred, in the current case 8 bits), the parity bit (that informs the receiver if data have changed during

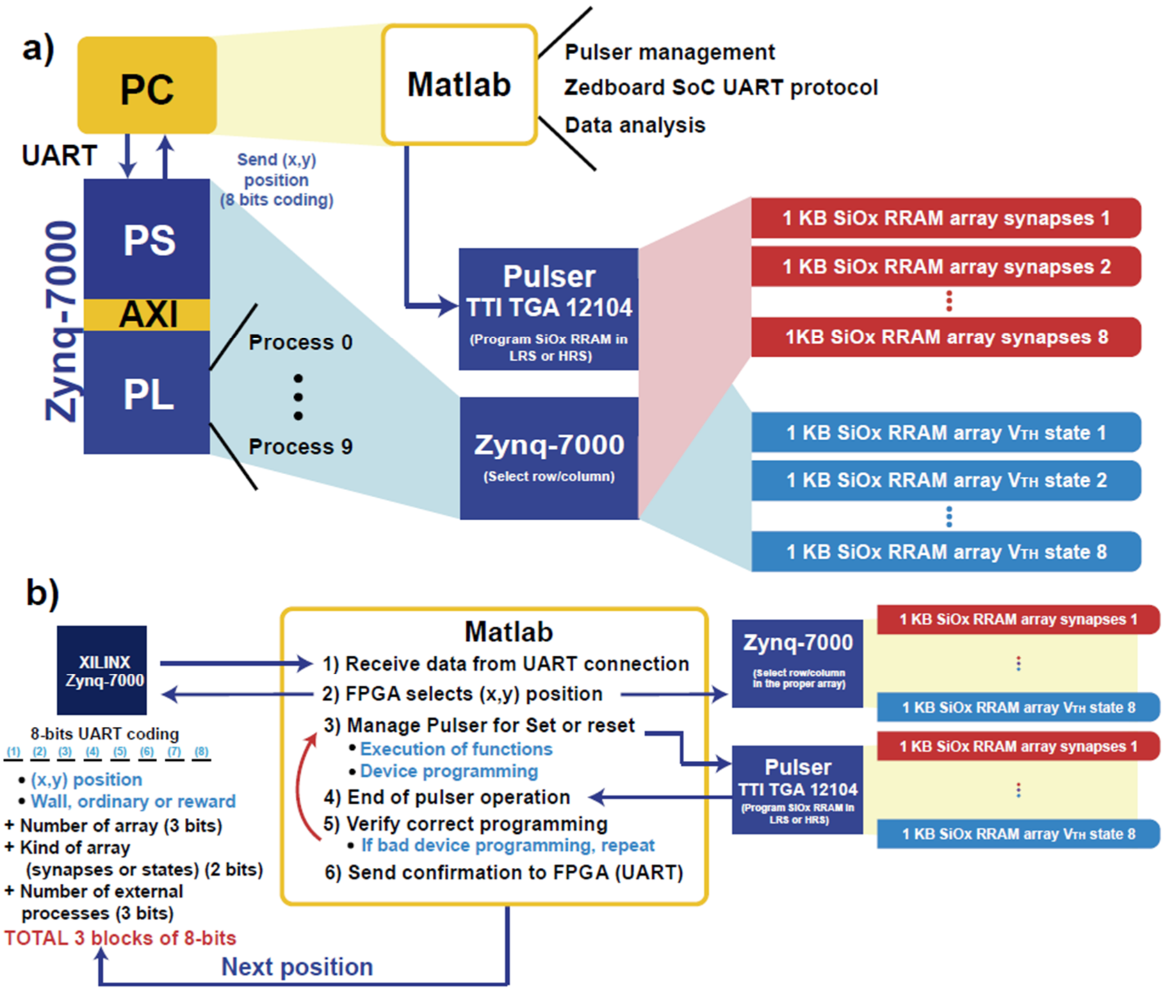

Supplementary Figure 6. Flowchart for the high-level description of the experimental setup. (a) General scheme for the connectivity of the different elements of the setup. The SoC communicates with MATLAB to manage the operation of the waveform generator TTI TGA 12104. This peripheral is connected to the TE, BE and gate of the 1T1R synapses. (b) Detailed description for the interface among the peripherals: a handshake protocol is implemented in order to ease the debug.

transmission) and the stop bit (equal to ‘0’). A baud rate of transmission of 230400 bps was used. The PC executes a MATLAB script that includes all the proper functions to configure the communication. Once the user has defined the initial data, the information is transferred from MATLAB to the SoC. In the Zedboard, the UART peripheral is connected to the PS part of the SoC. As the PL part is the one that performs the readout of the digital signals coming from the cardinal points (i.e. the neurons) and selects the row/column of the SiOx RRAM arrays, both PL and PS must communicate to each other. Thus, it has been used the AXI4 (Advanced eXtensible Interface) communication protocol which is part of the ARM AMBA (Advanced Microcontroller Bus Architecture) designed for on-chip communication and managements of functional blocks. The PS part of the SoC is programmed in C ++, with the pre-compiled functions for managing the UART. On the other hand, the PL part is configured by an FPGA. Therefore, the hardware description of the CLB (Configurable Logic Block) and of the DSP (Digital Signal Processor) elements has been carried out using standard VHDL language. A generic position (i, j) varies between 0 and 31, thus requiring 5 bits for coding the coordinates “i” and “j”, respectively. In addition, the SoC must know the role of that position: wall position, ordinary position or reward position. As the implemented UART communication transmits 8-uint (unsigned integer) bits of functional data, 2 packets are needed for transmitting one position. Supplementary Fig. 5 shows a block scheme related to the sending and receiving data with respect to the maze positions. The 8-bits of UART have been coded as follow: the first bit determines what kind of information is sent: “i” position (row) or “j” position (column). If the position “i” is sent, the second and the third bits determine the kind of position related to the row: a wall is coded as “00”, an ordinary position corresponds with “01” and the reward is coded as “10”. Otherwise, these bits are coded as “11”. Finally,

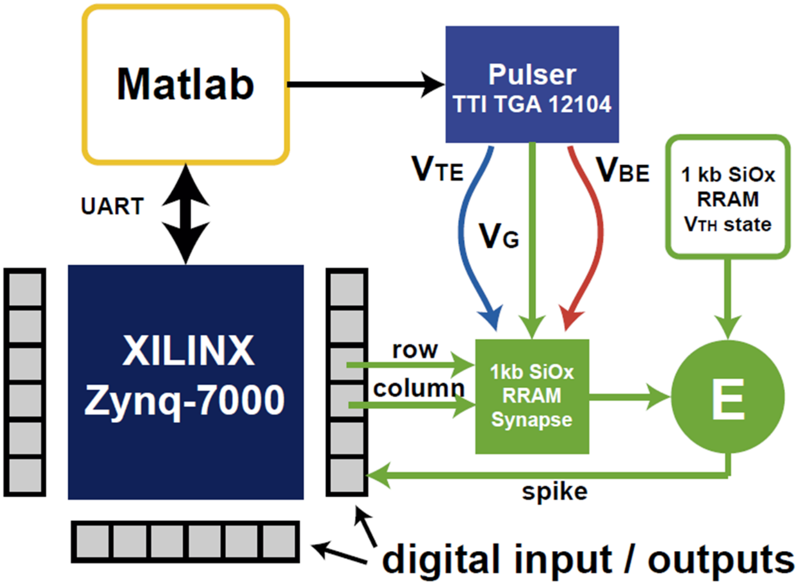

Supplementary Figure 7. Connectivity between the SoC, the array and the output neuron. The output neuron reads the current from the multiplication between V_{COM} and the conductance value of the selected 1T1R SiOx RRAM. The PL part of the SoC sends the row and column signals to the 1kb array and it receives back the spikes coming from the neuron. As the FPGA is managed by an internal clock at 50 MHz, a maximum integration time has been defined: if the neuron remains silent during this interval, the FPGA selects another cardinal position. If, on the other hand, the neuron spikes, the FPGA saves the delay. The winner is the neuron that spikes first.

bits from fourth to eighth represent the row and column values. These data are stored in registers within the SoC for commanding the external peripherals during the system operation and for debugging purposes.

System interconnections

As mentioned in the previous paragraph, the SoC acts as the master of the system: (i) it manages the programming phase of the cells through the UART communication; (ii) it defines the movement inside the maze selecting the proper position and synapse. Supplementary Fig. 6(a) shows a general scheme of the system. The Zynq-7000 communicates with MATLAB that rules the waveform generator in order to set, partially set, or reset the devices, as determined by the penalty/reward situation. A more detailed explanation is proposed in Supplementary Fig. 6(b). First of all, the SoC determines the analysed cell (row and column), and the data associated to this device: the “i” and “j” position, the array to which it belongs (enumerated from 0 to 7), the kind of array (synapses or states), the type of position (wall, ordinary or reward) and the number of the VHDL processes in execution. This information is coded and sent via UART to MATLAB. Note that the number of packets to be sent is 3 instead of 2: this is because the system must manage not only the devices that determine the movement towards the cardinal points (synaptic array), but also the devices that reduce or increment each neuronal threshold (state threshold array).

The programming condition of these memory elements is different from case to case: in the synaptic array, all the devices tend to be programmed in HRS or LRS due to Hebbian learning mechanism; on the other hand, in the state threshold array, the cells are initialized at a generic resistance value which is modulated as a function of the firing excitability of the device. During fire, in the case of ordinary positions, the state devices reduce gradually the LRS value by increasing I_C, i.e., they are incrementally set. However, if the analysed neuronal threshold position refers to a wall (penalty) or to the final goal (reward), this cell must be programmed in LRS or HRS, respectively. Thus, MATLAB receives 24 bits of coded data from the SoC with all the information and, depending on the selected position and the VHDL process that is currently executed, it defines the value of the variables (I_C, V_{STOP}, slope speed, …) that the waveform generator needs for the correct programming of the SiOx RRAM devices. The PL part selects the row and column of the proper array, and the TTI TGA 12104 applies the correct voltage to the TE, BE and gate of the 1T1R cells.

Using a verification algorithm, it is possible to determine if the device has been programmed in the desired resistance value. If not, the device is reprogrammed till it satisfies the constraint.

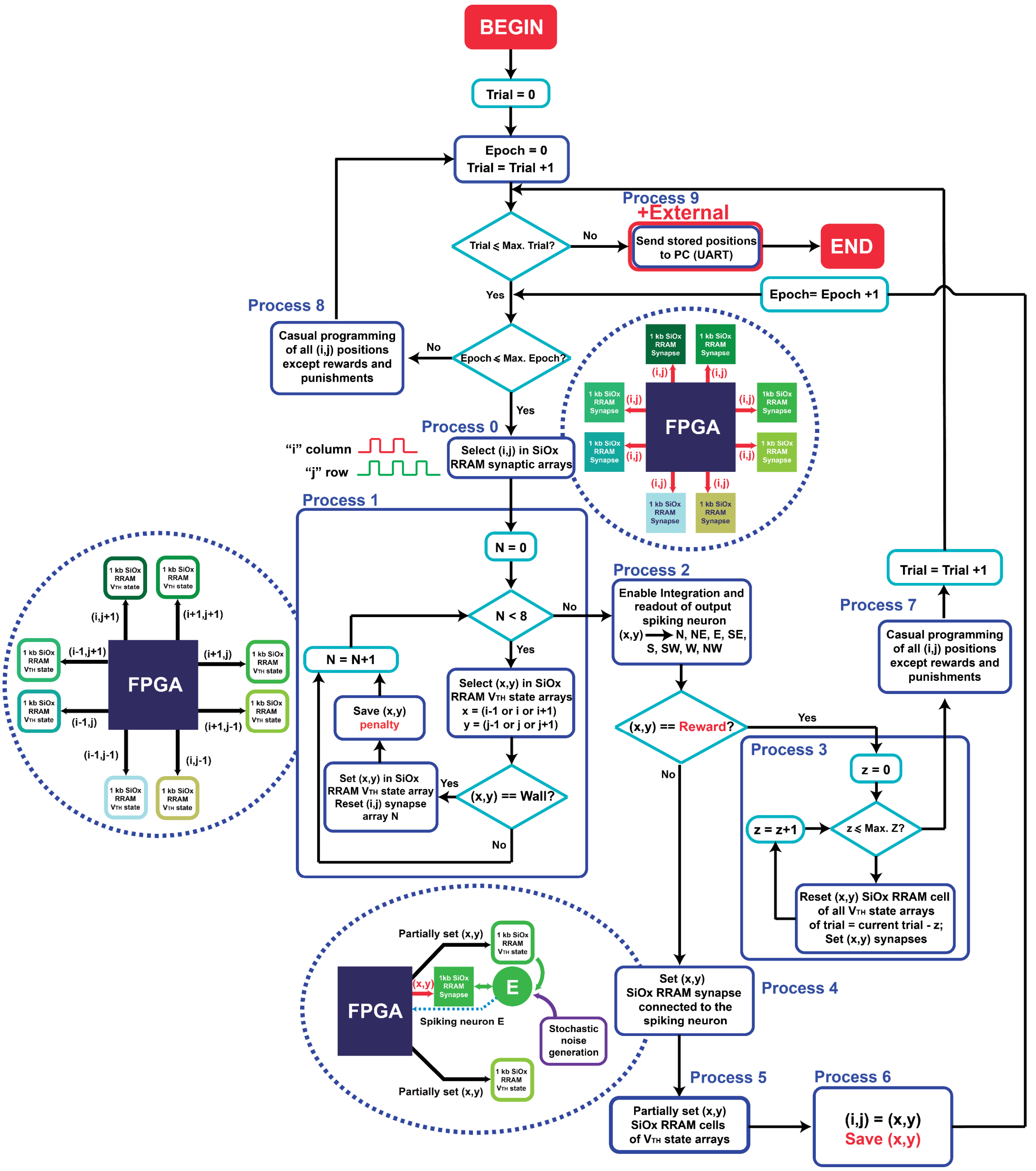

Supplementary Figure 8. Flow chart of the processes in the SoC. Most of the processes are performed in parallel, to reduce the operation time of the FPGA and efficiently exploit the parallel distributed resources. Note that the sources of stochasticity in the neural computation are also considered in the flow of the diagram. The "External" box refers to the possibility of debugging the hardware using the laboratory equipment such as the Oscilloscope or the Parameter Analyzer.

Once the memory element is correctly programmed, the SoC receives a confirmation from MATLAB, and it continues to execute the further processes. Supplementary Fig. 7 shows a scheme about the integration process of the current. The FPGA (PL part of the SoC) selects the 1T1R cell (row and column signals) of the SiO_x RRAM array. The TTI TGA 12104 waveform generator applies the correct voltages at the TE, BE and gate terminal, to correctly set or reset the cells. The FPGA sends in parallel the row and column signals to each "Synapse array" and "State threshold array". During the integration, the bottom, the gate, and the top electrodes share the same voltages since they are common to all the arrays. In addition, since the FPGA had previously selected in parallel the row and column of the State threshold array, each output neuron has the correct threshold voltage associated

to the corresponding cardinal position. Note that it would be also possible to apply all the memory-related signals using the SoC only but paying in terms of programming accuracy and debug efficiency.

The FPGA uses the 50 MHz internal clock of the Zedboard and reads in parallel the spiking signals coming from the cardinal neurons. From Monte Carlo and analytical simulations of the analogue integrator of the neurons, it is possible to determine the interval (number of clock pulses) in which the neuron should fire. The FPGA calculates the delay between the beginning of the integration and the firing for each neuron, and waits during the integration time, choosing as preferential direction the one connected to the neuron that has fired first.

The analogue signals that correspond to the integration and fire neurons are analysed by connecting the proper circuital nodes to the channels of the Tektronic MSO58 oscilloscope using triaxial cables. Statistical data can be also obtained from the information stored into the FPGA registers. Note that Vivado, the program used to manage the FPGA, gives a power consumption estimation of the SoC once the codes have been synthesized and implemented. The power consumption of the output neurons and arrays, on the other hand, can be computed by multiplying the constant value of the power supply by the delivered current.

Programming for signal management

In this section, the programming procedure for the PL part of the FPGA is described. In particular, this section gives a general overview about all the processes involved into the development of the reinforcement learning algorithm, Supplementary Fig. 8. The sequence of the operations is presented as a block flow diagram, and it follows the same scheme already presented for the Monte Carlo simulations in the first part of the technical discussion of this chapter. Furthermore, note that the proposed representation follows a digital description even for the analog parts, such as for the programming of the memory devices.

There are two types of processes which can be implemented in the PL: internal and external.

The internal deals with the operations performed within the SoC; the external communicates with MATLAB for programming the devices, and it is mainly related to the penalty and reward mechanisms. The flow chart begins with the definition of the number of trials and epochs within each trial. The FPGA knows the initial position and it sends the proper row and column signals to each one of the synaptic arrays connected to it (Process 0). In addition, it calculates the new cardinal position, starting from the initial one. The FPGA also selects the correct devices that determine the threshold voltage of the integrate and fire neurons, giving the penalty if any of the cardinal points correspond with a Wall of the maze (Process 1). As explained in the previous subsections, the SoC receives the spikes from the output neurons (Process 2). If the spiking neuron corresponds with a reward position, the trial finishes and the 10 latest positions receive the reward (Process 3), while the other positions are casually re-initialized (Process 7). If the spiking neuron corresponds with an ordinary position, the synaptic SiOx RRAM is set (Process 4) and the state device is partially set, thus increasing the threshold of that position (Process 5). The initial position is substituted by the cardinal position of the first spiking neuron, and it is then saved (Process 6). If the agent does not reach the final goal during the trial, the ordinary positions are casually reinitialized (the agent just remember penalties and reward from trial to trial). If the agent achieves the maximum number of trials, the data related to the positions are sent to MATLAB for debugging purposes. The source of stochastic noise is also considered in the computation. Furthermore, note that the “external” indication refers to the possibility of debugging the hardware results with the laboratory equipment, such as oscilloscopes or analysers.

TECHNICAL OVERVIEW OVER STDP LEARNING PARADIGM

In this section, we are going to provide further details about the STDP learning paradigm.

The pre-neuronal signal (which stands for the current position occupied by the agent) excites the gate of the selector of the synaptic RRAM element by sending a burst of rectangular pulses (2.2V of amplitude with duration 700ns). At the same time, the Top Electrode of the synaptic element is biased at a read voltage (which can range between 50 mV to 150 mV), thus driving the selector in ohmic state. The current, which depends on the state of the RRAM synapse, Fig. 1(b) of the manuscript, is integrated, as also reported in Fig. 2(b), and then compared to the internal threshold of the post-neuron, which is ruled by the corresponding "state" device, Fig. 2(a) and Fig. 2(d). If the threshold is overcome, a programming signal arises and directly potentiates the synaptic element acting on the top electrode of the synapse connecting the current position with the next firing neuron. At the same time, the neuronal threshold is correspondingly updated in order to keep trace of the fire excitability of the new spiking neuron (homeostatic mechanism), Fig. 2(a-d).

What we have just described takes into consideration a configuration in which the pre-neuron (the current position of the agent) excites the post-neuron, which eventually fires. However, biological studies demonstrate that the post-neuron could also fire before the pre-neuron, thus causing depression of the synaptic connection, middle picture in Fig. 2(a) of the manuscript[2]. This "time-dependence" (which, nevertheless, gives the name to the spike-timing-dependent plasticity), is mapped in hardware by using LFSRs (linear feedback shift registers) which randomly select neurons of the network to give rise to uncorrelated spiking activities. Note that the biological firing activity has been modelled as in Fig. 2(a), where also the refractory period and the depression signal are shown. In particular, when such depression signals happen in the nearest positions to the current one, the connecting synaptic element is reset (i.e., depressed) thanks to the negative polarity of the top electrode, as already demonstrated in previous works[3]. Furthermore, the reset signal starts with a "refractory period" of 1μs, as it happens in biological neurons[3]. Note that the generation of the pulsed programming signal can be managed internally by the hardware system or, conversely, by the pulser generator (refer to Supplementary Fig. 7-8) for debugging purposes or very precise programming of the synaptic weights.

Note that the integration of the synaptic current can be also done digitally without relying on the analog circuitry reported in Fig. 2 of the manuscript. This further method works as reported in the following: (i) the readout current from the synaptic elements feeds transimpedance amplifiers (OTAs) which translate the signal into the voltage domain; (ii) then, such voltage is sampled by an analog-to-digital converter and (iii) digitally integrated. Finally, (iv) the value is used in the computation with respect to the neuronal threshold and zeroed after the eventual firing activities. This method could be also useful for a better management of the STDP power consumption.

A precise definition of the synaptic weights requires not strictly necessary additional power consumption. In fact, differently from what reported in other works[4,5], the STDP algorithm can be also operated in a bistable way (i.e., switching the synapse from LRS to HRS and vice versa): in this way, the exponential model of the STDP is digitalized and the overall system requires less power consumption and area. The digitized STDP is not affecting the accuracy of the neural computation, as discussed in several works demonstrating the low-power neuromorphic computation based on the STDP algorithm[6,7]. On the other hand, the multilevel programming of the state device is fundamental for the homeostatic definition of the "state" device since it sets the internal threshold of each position and keeps trace of the neuronal excitability.

Related to this last consideration, note that the programming of the synaptic devices can be performed in two ways: (i) using the implemented circuits on the boards by means of the SoC Zync7000, or (ii) commanding the pulser and the oscilloscope (best option for debugging purposes), Supplementary Fig. 7.

DISCUSSION OVER THE RATE OF ENVIRONMENTAL DYNAMISM

One of the most interesting studies to carry out in this context is referred to the grade of resilience of the proposed hardware, with particular reference to the level of dynamism of the environment (i.e., rate of changing of the walls). The dynamism allows to verify the capability of the agent to remember the previous path to get the reward when, for instance, the maze dynamically modifies a wall in the midway of the optimum path. During the operation, an external user can decide the moment (i.e., the trial) in which the maze changes shape. In this situation, the SoC stops the operations and asks to MATLAB to send the new positions of the maze, in order to load a new environmental configuration. Then, the FPGA continues to operate, but in this case, some positions that initially were included as rewards are now treated as penalties. Thus, the agent must find another path towards the goal. This further activity of re-arrangement of the weights brings about an increased requirement of energy to get to the solution with respect to the lowest energy required for the exploration of a static maze, as shown in Supplementary Fig. 9.

The system tends to find the solution, whatever the shape of the environment is. However, the rate of changing walls can strongly affect the choices of the agent, also leading to non-optimum cases. In fact, if the rate is too high, the system self-adapts its structure to find a path that is likely to undergo, on average, the lower number of penalties, rather than looking for the fastest way to get to the reward as a function of each environmental situation that experiences.

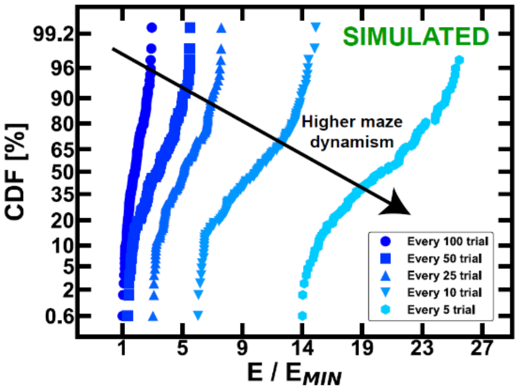

Supplementary Figure 9. Increase of the energy requirement as a function of the dynamism of the walls of the maze (change of the environmental configuration). In order to get to the final solution, an increase of the dynamism requires more time and more energy.

DISCUSSION OVER THE RRAM AND CMOS APPROACHES TO REINFORCEMENT LEARNING

In this section we propose a brief deepening over the “scalability” concept of artificial intelligent hardware.

Scalability of a neural network is achieved when the system can learn a concept rather than a pure mechanism (answering to questions like: what is a penalty? How can an artificial agent avoid it? How can the concept of the penalty be generalized? …).

In standard deep learning approaches, this concept-learning could be achieved by performing deep training of free trials of explorations which, however, require time and power expensive algorithms, as we also discuss in Section “Discussion” of the manuscript [8,9].

For instance, there have been evidences that if a problem is formulated as a partially observable Markov decision process within the actor-critic framework for the definition of the reward and penalty policies, it is possible to define a deep learning training approach capable of optimizing complex navigation tasks, such as navigation among obstacles[10]. By means of a recurrent deterministic policy gradient algorithm (called RDPG), Wang et al. demonstrated that it is possible to reach the 99.33% success in the navigation procedure. However, note that the training procedure for the deep learning approach requires, in the optimized fast-RDPG, 3000 training episodes, which is a far higher number with respect to what our neuromorphic recurrent network needs thanks to the homeostatic STDP also for a complex case such as the Mars Rover navigation test.

On the other hand, bio-inspired approaches with RRAM devices allow to map, by means of STDP-based Hebbian learning and homeostatic scaling, all the penalties and rewards that have been directly experienced by the agent. This is very relevant, since it means that the feature of the penalty, as well as the optimum behaviour to find a solution, is directly recorded in the hardware (faster and at lower power consumption, since the Von Neumann bottleneck is avoided thanks to the RRAM devices).

Such information is the core of the reinforcement learning procedure and it can be then processed in two ways:

1) Elaborating it by using deep learning approaches in CMOS in order to use the recorded information in standard neural networks partially avoiding the time and power consuming training procedures.
2) Modulating the information coming from unsupervised Hebbian learning, e.g., studying the shape of the penalties and/or of rewarded path and using it for transfer learning in new environments (see the main manuscript, subsection “Reconfigurability of the hardware”).

Indeed, the capability of continual online learning, is a key driver for several applications. This is the reason why there have been several attempts in building “meta-learners” using standard neural networks which try to use past information to overcome the expensive algorithms required by deep learning training procedures. In particular, a meta-learner regards the action of training a system on a distribution of similar tasks, in the expectation, for transfer learning, to catch the essence of the problem rather than a specific case study[11].

However, even considering these meta-approaches, several CNN-based training algorithms are anyway demonstrated to be necessary to provide the system with an optimum policy map for the required navigation task, falling again in the power and time bottleneck, and thus highlighting the benefits of a full bio-inspired approach to autonomous navigation with respect to such hybrid approaches. As an example, AlphaGo Fan, a software to play the game of “Go” required 1,920 central processing units (CPUs) and 280 graphics processing units (GPUs), consuming a peak power of half a megawatt. Application-specific integrated circuits could reduce power consumption, but the problem remains, since the majority of neural network parameters (for example, weights) are stored in dynamic random-access memory (DRAM): data are moved back-and-forth between the DRAM and the processing unit thus highlighting high latency and increased power consumption. The growing challenge of this communication bottleneck, together with the saturation of Moore’s law, limits the speed and the energetic efficiency of CMOS platforms for advanced reinforcement learning, thus highlighting, on the other hand, the relevance of bio-inspired algorithms based on non-volatile memories.

ADDITIONAL INSIGHTS OVER THE SCALABILITY TOPIC

In this section we are going to discuss the scalability of the hardware proposing three possible ways for the management of the computing architecture when the computing resources are limited.

1. *Rough solution* – It is possible to reduce the computational effort by providing lower sampling of the positions covered by the agent, meaning that a single step of the agent covers more physical space of the environment. This behaviour of the agent can be simply triggered by a CMOS-based counter which counts, during a first limited trial, the number of firing activities from penalty-to-penalty event, thus giving a rough estimation about the granularity of the sampling that can be applied to the research algorithm.

   However, this solution can be critical in terms of safety and reliability, since the system takes advantage of a rough estimation, and it is not fully reactive to the actual configuration of the environment.

2. *Medium solution* **-** The second way is related to the "paging" that is done in current processors. The main goal of this procedure is to bring and compress part of the information stored in the RAM to the Hard Disk Drive, thus improving the scalability of the computing resources. Such choice can be also implemented in RRAM-based systems, but it is not an optimum solution. Basically, the paging enables the use of the processor unit for the current computation while keeping the previous details stored in other CMOS-based memory units. This method has a major drawback related to the Von-Neumann bottleneck since data must be fetched not only between the computing unit and the DRAM memory, but also towards the main memory (hard disk), thus arising significant power consumption and time latency. Furthermore, the standard non-volatile memory elements are not built in the back-end-of-the-line, raising a problem in terms of area efficiency with respect to the bio-inspired hardware proposed in this work.

   Thus, even taking into consideration an abstraction of the stored information, the Von Neumann bottleneck limits the scalability of the resources. The problems related to the Von Neumann bottleneck are the ones that are currently hindering scalability in standard processors used for conventional deep learning algorithms[9].

3. *Best solution* **-** The third approach is based on the second one, but it proposes a further advancement which makes the bio-inspired approach completely scalable and far more efficient than the state-of-the-art.

   Basically, it is possible to take advantage of the RRAM-based matrices for relying on the in-memory computation and avoiding the Von-Neumann bottleneck.

   In particular, the memory array can be continually exploited by the bio-inspired computation until the complete memory resources are fully allocated. Then, when the memory array is completed, the direction of movement, the number of steps and some further information (such as the rewards) can be saved in separated registers (which can be also RRAM-based) as pure coordinates (strongly limiting the data fetch). Thus, the RRAM computing elements are practically ready again to perform the explorative trials, to abstract the previous maps of exploration and to reference the stored coordinates to the effective number of "refreshes" of the RRAM memory arrays.

   The same approach could be exploited both when the agent undergoes penalties and when it does not. In every case, the relevant points can be safely compressed by means of coordinates and mathematical representations of large areas, and the bio-inspired exploration can go on assuring the highest possible accuracy following the approach described in Fig. 5(e,f) of the manuscript.

   Thus, this third solution overcomes all the discussed points, and it also offers a valid strategy to get hardware scalability.

ADDITIONAL INSIGHTS OVER THE THEORETICAL MODELLING OF BIO-INSPIRED NETWORKS FOR REINFORCEMENT LEARNING

In the following we provide a mathematical relationship between the reinforcement and circuital parameters and the corresponding waveforms of the learning activity. In particular, we are going to provide further mathematical modelling referred to Equations IV and V of the main manuscript.

Reward and penalty function

The reward function acts on both the synaptic connection linking the positions "s - s'" and the internal state "s". When the agent undergoes a reward, the, internal state is driven to high resistive state, in order to lower the threshold, Fig. 3(h) of the manuscript, while the connecting synaptic element undergoes potentiation due to usual STDP evolution, Fig. 3(j). Conversely, if a penalty occurs, the internal state is brought to LRS abruptly, thus causing an increase of the threshold, Fig. 3(g) while the connecting synaptic element is depressed, Fig. 3(j). The depression of the synaptic element could be avoided, since the depression would have been anyway provided to these positions by the stochastic noise applied to the system with the LFSR registers used for accessing random neurons (refer to subsection "In-memory computing for autonomous navigation"). Thus, the reward function, for both state and synaptic elements, can be expressed by the following formulas:

$$R = \begin{cases} \frac{d\varphi}{dt} = Ae^{\frac{-E_A}{KT(z)}}\left(\frac{\varphi_{MAX}-\varphi}{\varphi_{MAX}}\right), for\ every\ synapse\ receiving\ a\ reward\ and\ state\ receiving\ a\ penalty \\ \frac{d\Delta}{dt} = Ae^{\frac{-E_A}{KT(z)}}, for\ every\ synapse\ receiving\ a\ penalty\ and\ state\ receiving\ a\ reward \\ G_{syn}(s,s') = \max\left(\frac{d\varphi}{dt}\right), LRS\ for\ all\ the\ rewarded\ synapses - Binary\ STDP \\ G_{syn}(s,s') = \max\left(\frac{d\Delta}{dt}\right), HRS\ for\ all\ the\ synapses\ undergoing\ penalty - Binary\ STDP \end{cases} \quad \text{(S\_I)}$$

Where:

- φ is the conductive filament diameter that grows inside the RRAM Silicon oxide following the Arrhenius law during each SET. In particular, the migration in the oxide of ionic defects is what rules the modulation of the resistive state of the RRAM[12]. In fact, the final multilevel resistance of the device depends on the number of defects brought in the device from the top electrode reservoir. Note also that the ionic defects are driven by the electric field due to the applied voltage pulses to the top electrode and thus to the risen temperature "T" along the cross-section of the device "z". The temperature profile can be obtained solving the 1-D steady state Fourier equation, which depends on the current density J which crosses the device[12]. The current J is chosen by setting the gate voltage V_G, Fig. 1, of the selector. Different values of J give rise to different values of T and to different values of R.

  Thus: the temperature dependence is directly related to the voltage applied to the top electrode of the RRAM device, Fig. 1(b), and to the gate of the RRAM selector, Fig. 1(c).

- φ_{MAX}: Maximum reachable filament diameter.

- Δ: the migration of the charges due to an inversed electric field generates a gap in the oxide, which increases the resistance. For this reset process the same physical laws apply as for the conductive filament φ, but with opposite polarity.

Note that the physics for the formation and disruption of the conductive filament is the same (only the driver of the electric field changes polarity, i.e., positive or negative voltages to the top electrode).

Furthermore, note that the analog increase or decrease of the resistance is directly proportional to the gradual creation and disruption of the conductive filament. Thus, the formulas are also useful to describe the homeostatic multilevel mechanism, Fig. 3(f) of the manuscript.

Synaptic evolution trial after trial

Those synapses which do not undergo the reward function, i.e., penalty or rewards, show a pure STDP tendency. For this reason, the proper description is time dependent.

The average synaptic evolution in time can be expressed by the following formula:

$$\frac{dG_{syn}}{dt} = AR_{PN}\left(G_{MAX} + G_{MIN} - 2G_{syn}\right) + C(G_{MAX} - G_{syn})(G_{syn} - \alpha N G_{MIN})(P - N)R_P \qquad \text{(S\_II)}$$

Where:

- A, C, α are fitting parameters.
- G_{syn} is the average conductance value.
- G_{MAX} and G_{MIN} are the maximum and minimum values of the synaptic conductance (LRS and HRS).
- P is a figure of merit expressing the density of pre-neuronal spikes at the 1T1R gates of the network.
- R_P is the frequency of pre-neuronal spikes at a specific 1T1R gate.
- R_{PN} refers to the uncorrelated neuronal fire rate activity which could rise to random fire activity (refer to section "In-memory computing for autonomous navigation").
- N is a figure of merit for the density of stochastic depression signal which can counteract the synaptic potentiation, on average. This is very relevant for matching the bio-inspired algorithm3, as we report in Section "In-memory computing for autonomous navigation".
- The first part of the equation basically reports the condition for which, if no correlated excitation happens (R_P = 0), i.e., the agent is not exploring the environment, the system does not evolve towards any valuable state (something like white noise condition) and the average synaptic evolution remains undefined.
- The second part of the equation describes the actual behaviour of the STDP during the reinforcement learning exploration: if the burst of the current neuronal activity is significant (P>N), then, on average, the synaptic elements of the walked paths tend to have a lower resistive value. On the other hand, if we increase the stochasticity of the algorithm (P<N) the system evolves towards a high resistive state (the exploration is not effective anymore). Thus, the stochasticity is relevant only to increase the performance when it is very localized in the network (for increasing the randomness of the explorative trials).
- This model is very general and can be also extended to pure unsupervised learning activities (e.g., for pattern learning).

Probability function

The probability function "P", which describes the probability of the quality of a certain position "s" for moving towards another state "s'", is dependent on the homeostatic-based STDP mechanism. Thus, since everything is related to the RRAM evolution in terms of resistance, the probability function is directly dependent on the physical parameters described in Eq. S_I. Furthermore, the probability function depends on the synaptic evolution, trial after trial, of the synaptic connection between state s and s', Eq. S_II and Fig.3(j).

Moreover, note that the assessment of the probability function is dependent on the current configuration of the system: the bio-inspired algorithm reacts as a function of it, and it provides the best behaviour for getting the final reward, Fig. 3.

$TD_{N,t}$

Since the formula depends only on Q factors and since the Q factor is completely determined by the aforementioned equations, also $TD_{N,t}(a, s)$ is completely determined.

COMPARISON OF THE RESILIENT PROPERTIES BETWEEN BIO-INSPIRED AND DEEP LEARNING APPROACHES

In order to test the resilience of the software-based reinforcement learning, we studied the impact of a continually changing environment on the performance of the reinforcement learning computation. In particular, in order to generalize this topic, we investigate here a simple theoretical approach for both the bio-inspired network proposed in this work and the standard software-based reported in[13].

The bio-inspired network, as we report in Section "Exploration, optimization and recall", is plastic, in the sense that it maps the fire activity, as well as the penalties and rewards, by means of the homeostatic STDP, Eq. IV. This point directly affects the behaviour of the agent, since the bio-inspired paradigm modifies on-line the synaptic connections correspondingly to the current configuration of the environment, as shown in Fig. 3 of the manuscript. Thus, an environmental change does not affect the correctness of the computation, provided that the environmental evolution is not too fast, Supplementary Fig. 9.

On the other hand, conventional software algorithms based on Q-learning techniques, generally rely on deep neural networks which require a big number of training data. Thus, if the data distribution changes, as it happens in reinforcement learning for adapting the agent's behaviour to a changing environment, the computation can be problematic since data samples are not independent to one another. In particular, Q-learning requires to map the state (M_{state}), the action (M_{action}) and the reward (M_{reward}) of each point of the maze in a matrix form which has the dimensions of the environment. In formula:

$$M = \begin{bmatrix} m_{1,1,k} & \cdots & m_{1,N,k} \\ \vdots & \ddots & \vdots \\ m_{N,1,k} & \cdots & m_{N,N,k} \end{bmatrix},$$

In which "N" represents the side dimension of the environment and k = 1, 2, 3, the state, the action and the reward information, respectively. Thus, the formula for defining the Q-value of a state can be described as follows:

$$Q(s, a) = Q(M_{state}, M_{action}, M_{reward})$$

The goal of deep Q-learning is to minimize the cost function of the neural computation at every iteration "i" of each trial. The cost function can be written in this form:

$$\varepsilon_{Loss} = \frac{1}{2}(y_i - Q_i(s', a', \gamma))^2$$

Where "y_i" is the target to achieve (maximum reward per each position) and $Q_i(s', a', \gamma)$ is the current Q-value matrix.

In this context, the Q-learning takes advantage of a convolutional neural network which looks for the best combination of synaptic weights γ in order to minimize the cost function (i.e., the CNN looks for that combination of synaptic weights which maximizes the Q values). In particular, the output of the neural computation depends on matrix M, since this matrix describes the overall characteristics of the environment to explore. Thus, calling y_i' the output of the neural network, the best combination of synaptic weights is computed using this formula:

$$y_i' = F_{activation}[(M_{state}, M_{action}, M_{reward}) * W_\gamma + b],$$

Where "b" is the bias of the neural computation and $F_{activation}$ is the neuronal activation function (such as the sigmoid or the ReLu). Thus, by means of convolutional steps (* operator), the CNN is able to find the best combination of synaptic weights γ in order to optimize the behaviour of the neural network for the reinforcement learning task.

This means that, in contrast with the bio-inspired approach where the computation relies on the live experience of the agent, for standard Q-learning the calculation of the best synaptic weights depends on the current configuration of the environment which directly affects the "M" matrices.

Thus, if after some trials the configuration of the environment changes, the synaptic weights carried out by the convolutional operation (which can be in the order of millions[14]) are not optimized for the new topology, driving the user to a full re-training of the network. This behaviour well resumes the stability-plasticity dilemma of standard neural networks, as reported in section "Introduction" [15].

It is possible to optimize the neural network adaptation, however without reaching the plastic features of bio-inspired neuromorphic networks. Furthermore, expensive (for time and power) re-trainings of the convolutional neural network are necessary for enabling more plastic behaviours in standard software-based Q-learning. A possible improvement of the standard approach would require the exploitation of the RRAM-based matrix-vector multiplication for computing the M matrix. In this case, the multiply and accumulate operation of standard processors can be significantly reduced. However, as stated in Fig. 4(f) and in Fig. 4(g), the bio-inspired approach intrinsically provides more computing performance and less power consumption. For instance, energy-expensive re-training algorithms would be anyway necessary for providing resilience to the system.

We can conclude that the bio-inspired solution is intrinsically more prone to adaptation with respect to standard approaches.

SUPPLEMENTARY REFERENCES

[1] Fremaux, N., Sprekeler, H. & Gerstner, W. Reinforcement learning using a continuous time actor-critic framework with spiking neurons. PLOS Computational Biology 9, 1–21 (2013).

[2] Folke, C. et al. Resilience thinking: integrating resilience, adaptability and transformability. Ecology and society 15, 20 (2010).

[3] Prezioso, M., Mahmoodi, M.R., Bayat, F.M. et al. Spike-timing-dependent plasticity learning of coincidence detection with passively integrated memristive circuits. Nat Commun 9, 5311 (2018).

[4] Panwar, Neeraj, Bipin Rajendran, and Udayan Ganguly. "Arbitrary spike time dependent plasticity (STDP) in memristor by analog waveform engineering." IEEE Electron Device Letters 38.6 (2017).

[5] Lu, Ke, et al. "Diverse spike-timing-dependent plasticity based on multilevel HfOx memristor for neuromorphic computing." Applied Physics A 124.6 (2018).

[6] Muñoz-Martin, I. et al. Hardware Implementation of PCM-Based Neurons with Self-Regulating Threshold for Homeostatic Scaling in Unsupervised Learning. In 2020 IEEE International Symposium on Circuits and Systems (ISCAS), 1-5 (2020).

[7] Muñoz-Martin, I. et al. Unsupervised learning to overcome catastrophic forgetting in neural networks. IEEE Journal on Exploratory Solid-State Computational Devices and Circuits 5, 58–66 (2019).

[8] Wang, Z., Li, C., Song, W. et al. Reinforcement learning with analogue memristor arrays. Nat Electron 2, 115–124 (2019).

[9] Ielmini, D., Wong, HS.P. In-memory computing with resistive switching devices. Nat Electron 1, 333–343 (2018).

[10] Wang, C., Wang, J., Shen., Y. & Zhang X. Autonomous Navigation of UAVs in Large-Scale Complex Environments: A Deep Reinforcement Learning Approach. IEEE Transactions on Vehicular Technology 68, 2124-2136 (2019).

[11] Mishra, N., Rohaninejad, M., Chen, M., Abbeel, P. A Simple Neural Attentive Meta-Learner. arXiv:1707.03141v3 (2018).

[12] Ambrogio S., et al. Analytical Modeling of Oxide-Based Bipolar Resistive Memories and Complementary Resistive Switches. Transactions on Electron Devices 61, 2378-2386, 2014.

[13] https://pypi.org/project/pyqlearning/

[14] Sandler, M., Howard, A.G., Zhu, M., Zhmoginov, A., & Chen, L. MobileNetV2: Inverted Residuals and Linear Bottlenecks. 2018 IEEE/CVF Conference on Computer Vision and Pattern Recognition, 4510-4520 (2018).

[15] Grossberg, S. Competitive learning: from interactive activation to adaptive resonance. Cognitive science 11, 23–63 (1987).
