# Supplementary material for: A self-adaptive hardware with resistive switching synapses for experience-based neurocomputing
Source: Nat Commun. 2023 Mar 21;14:1565. doi: 10.1038/s41467-023-37097-5 (PMC10030830; doi:10.1038/s41467-023-37097-5)
Supplement: Supplementary file 3 — Description of Additional Supplementary Files [file 41467_2023_37097_MOESM3_ESM.pdf]

Description of Additional Supplementary Files

File Name: Supplementary Movie 1
Description: Illustration of the experimental setup and demonstration of the exploration of a dynamically-evolving environment via reinforcement learning
